# Orientation Relationships in Al_0.7_CoCrFeNi High-Entropy Alloy

**DOI:** 10.1017/S1431927617012442

**Published:** 2017-08-15

**Authors:** Leo T.H. de Jeer, Václav Ocelík, Jeff T.M. De Hosson

**Affiliations:** Department of Applied Physics, Zernike Institute for Advanced Materials, University of Groningen, Nijenborgh 4, 9747 AG Groningen, The Netherlands

**Keywords:** electron backscatter diffraction, orientation relationships, high entropy alloy, nucleation, phase transformation

## Abstract

A detailed microstructural evaluation was executed on the crystallographic texture as well as the mechanisms for nucleation, phase transformation, and grain growth in a Al_0.7_CoCrFeNi high-entropy alloy. The microstructure and crystallographic orientations were characterized by electron backscatter diffraction, and the chemical composition variations by energy-dispersive X-ray spectroscopy. The cast Al_0.7_CoCrFeNi alloy started in the BCC phase and partially transformed into the FCC phase. It was found that the Pitsch orientation relationship (OR) dominates the nucleation mechanism of the FCC phase; however, deviations with respect to the Pitsch OR are observed and are attributed to the differently sized atoms forming an ordered B2 phase in the alloy causing lattice distortions. The dual phase BCC–FCC microstructure contains FCC Widmanstätten plates oriented parallel to the {110}_BCC_ planes of the parent grain. It was found that the crystal orientation distribution after the BCC–FCC phase transformation is confined and is explained as a product of the governing mechanisms.

## Introduction

The advent of high-entropy alloys (HEAs) as introduced by Yeh et al. (2004) has given an impetus to the design of multicomponent alloy systems, which are particularly mechanically stable at elevated temperatures. HEAs are defined as alloys containing five or more principal elements with about equivalent atomic percentages so as to maximize the effect of the configurational entropy contribution to the total Gibbs energy of mixing. In recent years considerable attention has been paid to the microstructure, alloy preparation, and mechanical performance of various HEAs (Zhang et al., 2014). This interest was caused by the extraordinary properties of HEAs compared with their principal elements and other known alloys. A wide range of combinations of principal elements has already been explored in the literature (Maulik et al., 2016; Rao et al., 2016; Wang et al., 2016). One of the more popular combination of elements is AlCoCrFeNi, in which the concentration of one of the precursor elements is varied (Kao et al., 2009; Munitz et al., 2016; Zhang et al., 2016a, 2016b, 2016c). Despite the rather extended literature dealing with processing, almost no detailed work has been reported concentrating on the crystallographic orientation relationships (ORs) and distributions in multiphase HEAs. Needless to say, to fully understand the structure–property–performance relationships detailed information about the OR, which influences the materials’ microstructure, is a critical issue and of major concern.

An attractive feature of this particular alloy is that it consists of both the FCC phase and BCC phase. For Al_*x*_CoCrFeNi this multiphase character exists for the range 0.45≤*x*≤0.88 (Kao et al., 2009) and it provides the possibility to examine the OR between the BCC phase and FCC phase in detail. The absence of other phases makes the material suitable to investigate the OR in relation to the nucleation and growth of these two phases. The investigation of the OR in this material leads to the set of classical possibilities resulting in the generally known rational ORs of Bain (1924), Pitsch (1959), Kurdjumov–Sachs (K–S) (Kurdjumow & Sachs, 1930), and Nishiyama–Wassermann (N–W) (Wassermann, 1933; Nishiyama, 1934). Stimulated by various applications, research has been carried out on these ORs; i.e., not in HEAs but rather in other material systems ranging from relatively simple binary systems (Gotoh et al., 1988; Wang et al., 2014) to complex martensitic steels (Zhang & Kelly, 1998; Redjaïmia & Metauer, 2001; Verbeken et al., 2009; Cayron, 2014; Grewal et al., 2016). Only a single study on the OR in HEA can be found, which was, in fact, recently published by the current authors (Rao et al., 2016) using transmission electron microscopy (TEM), yielding only very local information about the existing OR between the BCC phase and FCC phase.

In contrast to conventional TEM, in this paper, we use the strength of electron backscatter diffraction (EBSD). The combination of EBSD and scanning electron microscopy (SEM) allows us to collect a large statistical sampling of phase boundaries to determine the BCC–FCC OR distribution found in Al_0.7_CoCrFeNi. In particular, during phase transformation of this alloy the FCC phase nucleates within the BCC phase grains, providing the possibility to observe the OR of the BCC to FCC transformation in detail, which is less frequently discussed in literature than the reverse transformation; i.e., of the transformation from the FCC phase to the BCC phase. In this study, we identified a set of mechanisms that determine the final microstructure and crystal orientation distribution after this BCC to FCC phase transformation.

## Concise Background of OR of BCC to FCC

Considerable work has been performed on the characterization of the OR between the BCC phase and FCC phase from an experimental, a computational, and a theoretical point of view in other materials than HEAs. This field started with Bain in the mid-1920s, characterizing the OR after the martensitic transformation, resulting in the Bain OR (Christian, 1975). The most commonly used ORs to describe the BCC–FCC interface (also called *α*/*γ* interface) are the Bain (1924), Pitsch (1959), K–S (Kurdjumow & Sachs, 1930), N–W (Wassermann, 1933; Nishiyama, 1934), and the Greninger–Troiano (G–T) (Greninger & Troiano, 1949) ORs. The experimental observations are mainly based on the FCC to BCC phase transformation; i.e., an FCC parent phase with a BCC daughter phase. The Bain, Pitsch, K–S, and N–W ORs are called rational ORs because of the low Miller index numbers for describing the common planes and directions; the G–T is called an irrational OR because of the high Miller index numbers. The description of an OR encompasses a pair of parallel crystal planes and a pair of parallel crystal directions for the parent phase and daughter phase. Table 1 provides the details for the FCC to BCC phase transformation. To date, most interfaces after phase transformation can still be characterized with the aforementioned fundamental ORs discovered in the first half of the 20th century.

**Table 1:** Conditions for the Parallel Crystal Planes and Crystal Directions, Which Define the Bain, K–S, N–W, Pitsch, and G–T OR for a FCC to BCC Phase Transformation (Verbeken et al., 2009).

OR	Plane	Direction
Bain	{100}_FCC_ ||{100}_BCC_	<100>_FCC_ ||<110>_BCC_
K–S	{111}_FCC_ ||{110}_BCC_	<110>_FCC_ ||<111>_BCC_
N–W	{111}_FCC_ ||{110}_BCC_	<011>_FCC_ ||<001>_BCC_
Pitsch	{100}_FCC_ ||{110}_BCC_	<110>_FCC_ ||<111>_BCC_
G–T	{111}_FCC_ ||{110}_BCC_	<123>_FCC_ ||<133>_BCC_

K–S, Kurdjumov–Sachs; N–W, Nishiyama–Wassermann; G–T, Greninger–Troiano; OR, orientation relationship.

The mathematical difference between an FCC to BCC OR and a BCC to FCC OR lies in the rotation description. It has been shown that the rotation from an FCC to BCC lattice is the inverse to the rotation from a BCC to FCC lattice (Verbeken et al., 2009). In the following it is relevant to recall that each crystal orientation of a cubic structure can be obtained by three subsequent rotations. The corresponding angles are called Euler angles (Kocks et al., 1998). Therefore, an OR variant can be described as a set of Euler angles. In Table 2 the Euler angles (Bunge notation) for one variant of the various ORs are shown for the BCC to FCC phase transformation and vice versa. The inversion of the rotation is obtained by interchanging the *φ*_1_ and *φ*_2_ Euler angles. For that reason, the descriptions of the Bain and K–S ORs are exactly the same for both directions of the phase transformation; however, for the Pitsch and N–W ORs the descriptions are interchanged. The N–W OR for the BCC to FCC phase transformation gives exactly the same rotation as the Pitsch OR for the FCC to BCC phase transformation and vice versa. A set of three Euler angles can be treated as a three-dimensional (3D) Cartesian coordinate and, therefore, all orientations of all grains can be plotted as a point in the Cartesian Euler space.

**Table 2:** Euler Angles Describing the Rotation from the Parent Grain Orientation to an OR Variant for the FCC to BCC and BCC to FCC Phase Transformation for the Bain, K–S, N–W, and Pitsch OR (Nolze, 2008).

	FCC → BCC			BCC → FCC		
OR	*φ*_1_ (°)	Φ (°)	*φ*_2_ (°)	*φ*_1_ (°)	Φ (°)	*φ*_2_ (°)
Bain	0	45	0	0	45	0
K–S	5.77	48.19	5.77	5.77	48.19	5.77
N–W	9.74	45	0	0	45	9.74
Pitsch	0	45	9.74	9.74	45	0

K–S, Kurdjumov–Sachs; N–W, Nishiyama–Wassermann; OR, orientation relationship.

It is interesting to note that the FCC to BCC ORs are extensively studied in meteorites, because meteorites form the BCC kamacite phase within the FCC taenite phase. Various meteorites with particular microstructures and ORs are reported in the literature. Fe–Ni meteorites are studied using different methods, including synchrotron diffraction and EBSD (Bunge et al., 2003; Nolze & Geist, 2004; He et al., 2006; Goldstein & Michael, 2006; Yang et al., 2011). In these cases, a strong K–S and N–W OR were found, as was a continuous range of orientations between the two ORs. The precipitation of BCC Widmanstätten cementite in an FCC austenite matrix was studied, in which a strong Pitsch OR was determined; however, Zhang & Kelly (1998) found in TEM experiments an OR that does not fit into the known rational ORs, but the Pitsch OR was not found for Widmanstätten cementite plate formation in an austenite matrix in their study. In two transformation induced plasticity steels for the transformation from austenite to bainite a strong K–S OR was detected, but N–W and Pitch ORs were also present (Verbeken et al., 2009).

## Methods and Materials

The Al_0.7_CoCrFeNi alloy was synthesized by the arc melting method in an in-house home-made electric arc-furnace. The high-purity precursor principal metals were melted in a protective Ar gas environment in a vacuum of 10^−6^ bar after pumping. After melting and mixing, the alloy was cast and cooled at a high cooling rate of about 100 K/s. No post-heat-treatment was applied. The cast alloy button with a mass of about 10 g was prepared for EBSD analysis by mechanical polishing and finished with a 0.04 *µ*m sized polishing agent.

A field emission gun-SEM (TESCAN, Brno, Czech Republic) in combination with an EBSD system (Edax Inc., Draper, UT, USA) was used for the crystallographic and microstructural characterization and an energy-dispersive X-ray spectroscopy (EDS) system (Edax Inc.) was used for the elemental characterization of this sample. To determine the crystallographic orientations with high accuracy, we used a large number of detected Kikuchi bands (10–12) for the indexing of the BCC and FCC phases, and we used the maximum resolution (no binning) of the Hikari Super EBSD (Edax Inc.) detector. The EBSD step size (500 nm) in a hexagonal grid was adjusted to fulfill the condition of having more than 1,000 points per FCC variant (Cayron, 2013).

The EBSD data were analyzed using the orientation imaging microscopy (OIM) Analysis 7.3 software and the MTEX MATLAB Toolbox for Quantitative Texture Analysis (Bachmann et al., 2010). The studied HEA provides very good Kikuchi patterns for 20 kV with 100% of the points being indexed. Nonetheless, a data-cleaning procedure was used to increase the probability for correct indexing. In the first step, confidence index (CI) standardization was realized with a grain tolerance of 5° and a minimum grain size of 5 pixels in at least two rows. This cleaning step does not change the detected phase or crystal orientation, it just equalizes the probability of correct indexing for all points in one grain to its maximum. In the second cleaning step, the orientation of points with a low CI(CI<0.1) have been modified to the orientation defined by the majority of its neighboring points with a good CI. No more than 2.5% of the scanned points have been modified by this cleaning procedure; moreover, in the presented OIM results only points with a CI>0.1 are shown.

## Results

**Figure 1:**
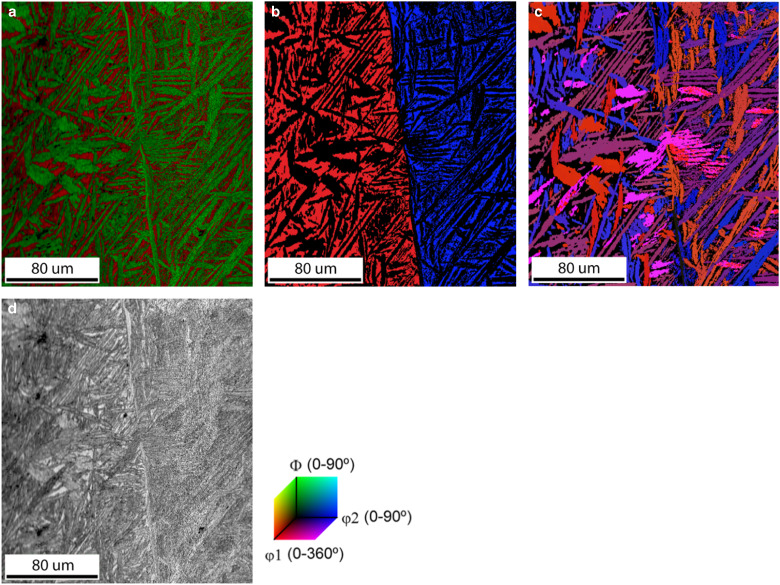
Representative microstructure of Al_0.7_CoCrFeNi high-entropy alloy after casting. **a:** The combined phase with image quality map, with the BCC phase in red and the FCC phase in green, shows the distinct morphology of the two phases. **b:** The Euler color map of the BCC phase shows that the map consists of two BCC parent grains. **c:** The Euler color map of the FCC phase shows the variety and limited possibilities in orientations of the FCC daughter plates inside these two BCC grains because of an orientation relationship. **d:** Image quality map shows the general microstructure.

After quenching, the very distinct and particular microstructure found in the Al_0.7_CoCrFeNi HEA has a Widmanstätten pattern; see Figure 1. This pattern has a clear ribbon-shaped structure that is commonly found in Fe–Ni-based meteorites (Bunge et al., 2003; Nolze & Geist, 2004; Goldstein & Michael, 2006; Yang et al., 2011) and in steel (Grewal et al., 2016). This HEA has a microstructure consisting of a mixture of the BCC phase and FCC phase. The BCC parent phase is shown in Figure 1a in red color and the FCC daughter phase in green color. The BCC phase and FCC phase have a surface fraction of 34.6 and 65.4%, respectively. In Figure 1 two BCC parent grains are observed, which are shown in the Euler angle map for the BCC phase in Figure 1b. The Widmanstätten pattern is formed by the FCC phase daughter grains. The daughter grains have different crystallographic orientations, which are shown in the Euler angle map for the FCC phase in Figure 1c. The image quality map in Figure 1d shows the general microstructure of the HEA. In this case we have two BCC matrix grains and many FCC grains originating from these two parent grains. In the cross-section, the observed FCC daughter grains are all ribbon shaped. The same microstructural picture is observed for many other BCC parent grains and, therefore, it is easy to conclude that the FCC grains must have a plate-like shape. The chemical composition differs slightly between the BCC phase and FCC phase; see Table 3. The BCC phase contains relatively more Al and Cr, and the FCC phase contains relatively more Fe, Co, and Ni. This means that the slightly heavier atoms have a larger concentration in the FCC phase than the lighter atoms.

**Table 3:** The Overall Elemental Composition of the High-Entropy Alloy and the Elemental Composition of the BCC and FCC Phase, Separately.

	Average		BCC		FCC	
Elements	at%	Error (at%)	at%	Error (at%)	at%	Error (at%)
Al	12.8	0.7	14.3	0.3	12.0	0.3
Cr	22.3	0.5	23.7	0.2	21.9	0.2
Fe	22.0	0.5	21.7	0.2	22.4	0.2
Co	22.9	0.5	20.9	0.2	22.4	0.2
Ni	21.0	0.5	19.4	0.2	21.3	0.2

**Figure 2:**
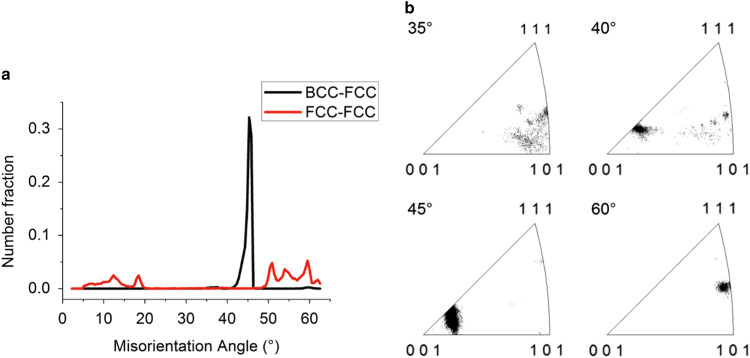
Misorientation characterization. **a:** The misorientation angle distribution between the BCC and FCC phases (black) with a peak at 45.7° and the misorientation angle distribution between FCC phase plates (red). **b:** Misorientation angle-axis distribution between the BCC and FCC phases for a given angle ±2.5° shows that the common rotation axis lies near the <001> crystal direction for misorientation angles close to 45°.

The distribution of the crystallographic misorientation angle between the BCC and FCC grains, shown in Figure 2, indicates that a strong OR is present. The misorientation angle distribution between the BCC and FCC grains shows a narrow distribution between 40.0 and 46.5° with a peak at 45.7°; see Figure 2a. The misorientation axis distribution lies mainly near the <001> crystal direction; see Figure 2b. This misorientation distribution corresponds to the Pitsch and K–S OR, which have a misorientation axis/angle of <0.08 0.20 0.98>/45.98° and <0.97 0.18 0.18>/42.85°, respectively (Verbeken et al., 2009).

**Figure 3:**
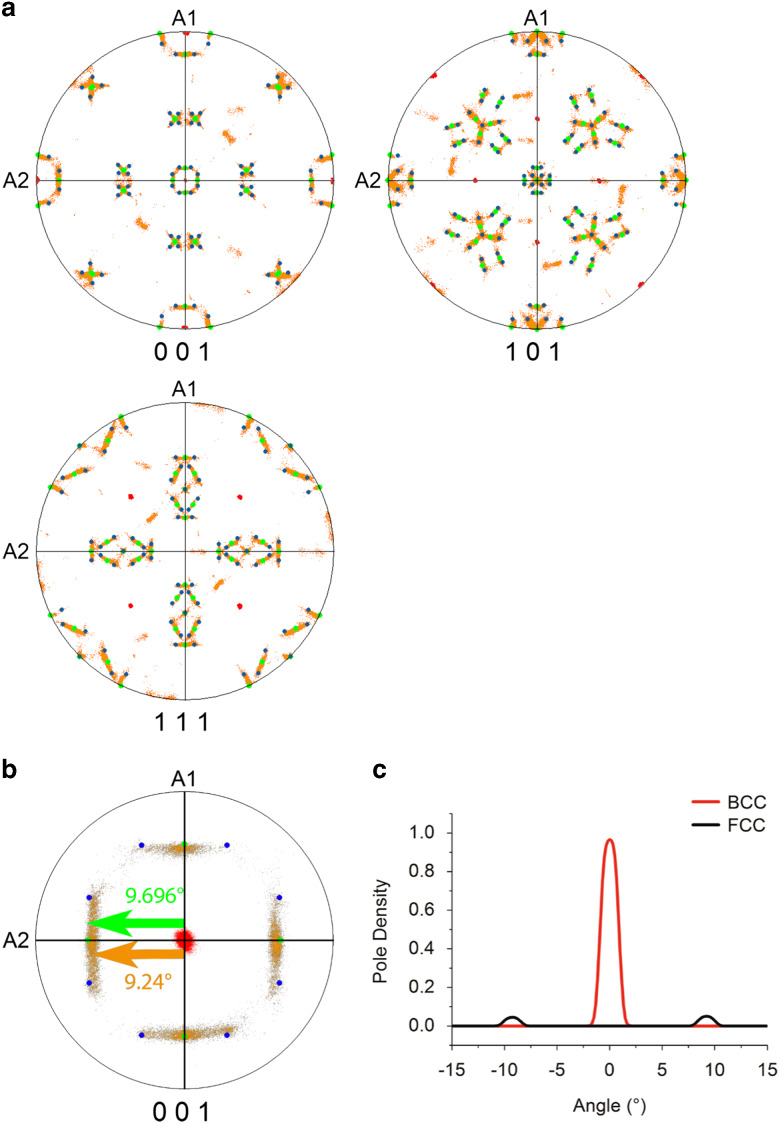
Orientation relationship in the high-entropy alloy (**a**) {001}, {011}, {111} pole figures show the BCC parent phase (red) and the agreement between the FCC daughter phase (orange), the Pitsch orientation relationship (OR) (green), and the Kurdjumov–Sachs OR (blue). **b:** {001} Pole figure magnified with a maximum polar angle of 15°. The theoretical angular distance between the center and the Pitsch variant is 9.696° and the experimental angular distance between the center and the center of {001} pole distribution along A2 is 9.24±0.01°. The deviation, therefore, is 0.46°±0.01°. **c:** Pole plot distributions in vicinity of the central point. The peak for the pole density of the BCC phase has a full-width half-maximum (FWHM) of 2.00°; the peak for the pole density of the FCC phase has a FWHM of 1.83°.

The crystal orientation distribution displayed by the pole figures presented in Figure 3 indicates which variants of the Pitsch and K–S ORs are present. The data shown in Figure 3 correspond to the parent grain on the right-hand side of Figure 1 and its daughter grains. The data were rotated in such a way that the main poles of {001}_BCC_ correspond with the directions of sample axes A1, A2, and A3. The BCC parent grain (in red), the FCC daughter plates (in orange), as well as the theoretical orientations of the Pitsch (in green) and K–S variants (in blue) are presented in the pole figures shown in Figure 3a. The experimental data show a clear overlap with the theoretical Pitch and K–S variants and also show a continuous points distribution between the Pitsch and K–S variants. It is also clear that the choice of the variant is random; no preference for one of the 12 Pitsch or 24 K–S variants is observed. To emphasize the experimental spread in the pole distribution, the center of the {001} pole figure is magnified to a pole figure with a maximum polar angle of 15.0° in Figure 3b. The distribution of the BCC and FCC pole densities along the A2 axis are given in Figure 3c. For the BCC and FCC pole density a spread with a full-width half-maximum (FWHM) of 2.00 and 1.83° are found, respectively. The expected precision of the EBSD system is estimated around 0.5° (Wright et al., 2011) and, therefore, a pole distribution with a FWHM of around 1.0° is expected. A more precise estimation of the experimental error was made by examining the distribution of correlated point to point misorientations inside one BCC grain, which results in an average misorientation of 0.36°. A similar estimation for the FCC phase was not possible due to the presence of a much higher local misorientation gradient. As the FWHM of the BCC pole density is wider than the 1.0° expected due to experimental error, the BCC grains must contain a small variation in lattice orientation, as was confirmed by grain reference orientation deviation map (not shown here). Therefore, a spread in the FCC poles is also expected. In spite of the observed overlap between the poles of the data and the theoretical OR variants, a small difference is still observed, which is well visible in Figure 3b. The centers of the {100}_FCC_ pole distributions do not correspond exactly with the {100} poles of the theoretical Pitsch and K–S variants. The measured difference between the pole of the theoretical Pitsch variants and the centers of the experimental pole distribution is 0.46±0.01°.

**Figure 4:**
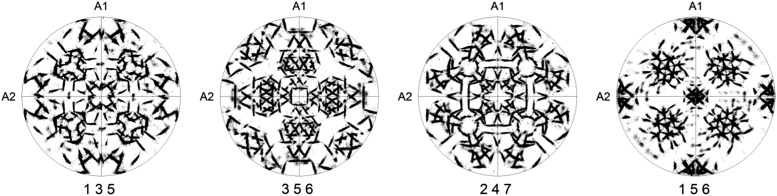
The four high-index pole figures of FCC phase show the orientation relationship. The {001} poles of the BCC phase in coincidence with sample axes A1, A2, and A3.

Due to symmetry, the low-index pole figures are not always suitable to study the OR due to overlap of poles and, therefore, the use of pole figures with high-index lattice planes has been suggested (Nolze, 2006). Figure 4 shows a few high-index number pole figures of the same experimental data as in Figure 3. Excellent distinction between the different poles of the FCC phase is evident, which means there is almost no overlap effect of multiple narrow peaks; however, a comparison of these high-index pole figures with pole figures predicted for individual ORs does not provide a clear answer about which OR is predominant in our sample.

**Figure 5:**
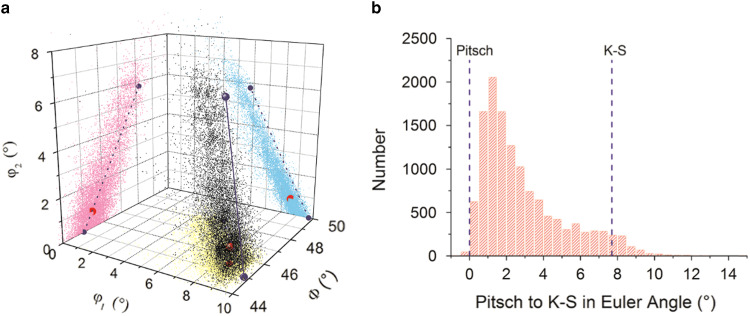
**a:** A three-dimensional view of the Euler subspace in Bunge notation (0°<*φ*_1_<10°, 44°<Φ<50°, 0°<*φ*_2_<8°) to determine the orientation relationships (ORs). This view shows the experimental data cloud (black) and the projection of the data cloud on the three surfaces (*φ*_1_=0°, Φ=50°, and *φ*_2_=0). Depicted in dark blue are the theoretical Pitsch (9.74°, 45.0°, 0.0°) and Kurdjumov–Sachs (K–S) (5.77°, 48.19°, 5.77°) ORs. The data cloud shows a good match with the dashed dark-blue line connecting the theoretical Pitsch and K–S OR. The local maximum is depicted in red (8.58°, 45.45°, 0.75°). **b:** Distribution of the orientations along the Pitsch to K–S line (dark-blue line). For this, the data have been projected on the Pitsch to K–S line. A majority of the points have a near-Pitsch variant orientation. The peak is found at 1.25°.

A different presentation of the orientation spread is possible in the Euler subspace; Figure 5. To study the orientation distribution of the FCC phase, we take a particular subspace in the Euler space and, therefore, a confined set of orientations in the vicinity of the theoretical Pitsch and K–S variants. The crystal orientations in Euler angles (Bunge notation) of the FCC plates (black points), the local density maximum (red), and the position of the Pitsch and K–S variant pair (blue spheres) are plotted in a 3D Cartesian space in Figure 5a. For clarity, we also plotted both the data and OR variants in Figure 5a as projections on each of the three side planes with *φ*_1_=0°, Φ=50^o^, and *φ*_2_=0°, respectively. It is clearly visible that the experimental data are spread along the Pitsch to K–S line; however, a small difference between the orientation distribution and the Pitsch to K–S line is observable. The maximum of the orientation density (*φ*_1_=8.58°, Φ=45.45°, *φ*_2_=0.75°) lies in the vicinity of the Pitsch variant (*φ*_1_=9.74°, Φ=45°, *φ*_2_=0°); see Table 4. To quantify the experimental orientation distribution between the Pitsch and K–S variant, the data points were projected onto the line connecting the Pitch and K–S OR in the Euler space. The density of this projection is depicted in Figure 5b. Figure 5 clearly demonstrates a strong Pitsch OR with a clear confined set of orientations along the Pitsch to K–S line.

**Table 4:** Orientations of the Theoretical Pitsch and K–S Variant and the Local Maximum of the Given Euler Subspace in Euler Angles (Bunge Notation).

OR	*φ*_1_ (°)	Φ (°)	*φ*_2_ (°)
Theoretical			
Pitsch	9.74	45	0
K–S	5.77	48.19	5.77
Local maximum	8.58	45.45	0.75

K–S, Kurdjumov–Sachs; OR, orientation relationship.

**Figure 6:**
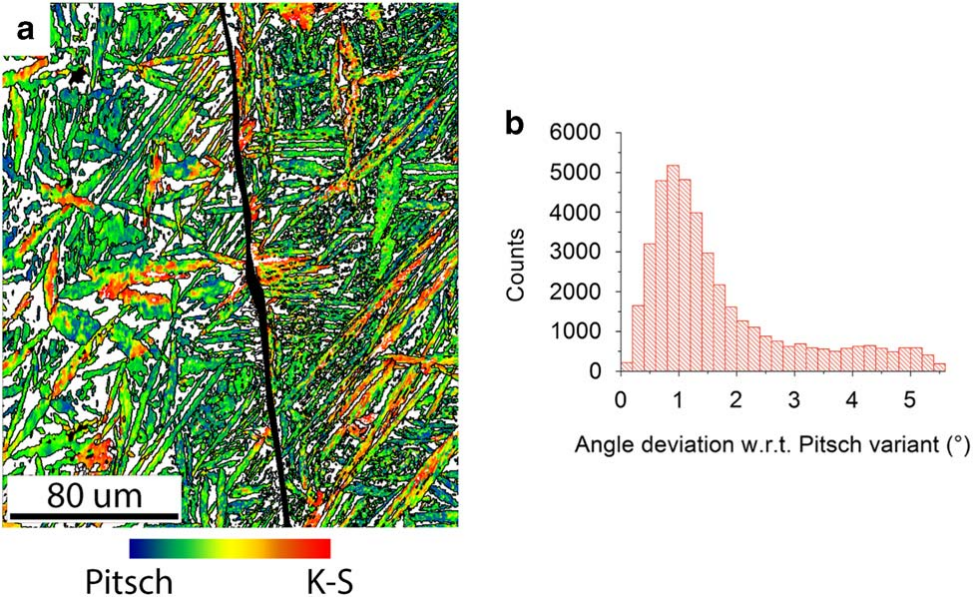
Distribution of Pitsch to Kurdjumov–Sachs (K–S) orientation relationship (OR) continuum through the sample. **a:** The orientation map of the FCC grains shows which areas have a Pitsch orientation (blue), which areas have a K–S orientation (red), and which areas have an orientation between a Pitsch and K–S orientation with respect to the average orientation of their parent BCC grain. The grain boundary between the two BCC grains has been highlighted (black). **b:** The misorientation distribution with respect to the nearest theoretical Pitsch variant. The theoretical K–S variants have a 5.26° misorientation with respect to the nearest Pitsch variant. The misorientation peak is found at 0.88°.

The deviation of the orientations with respect to the nearest Pitsch variant in the microstructure of the FCC phase is visualized in an OR map as shown in Figure 6a. This map was constructed as a crystal orientation map in which all 12 Pitch variants were included as a reference orientation with a maximum misorientation angle of 5.5°. The Pitsch variant orientations were calculated on the basis of the average crystal orientation of the BCC parent grain. All FCC orientations lie between a Pitsch and K–S variant, with the K–S variant having a misorientation of 5.26° with respect to its nearest Pitsch variant (Dahmen, 1982). In this way, the continuum of orientations between the Pitsch (blue) and K–S (red) ORs is represented. The OR map makes clearly visible how the OR continuum is distributed within this sample. The distribution of the misorientation with respect to the nearest Pitsch variant is displayed in Figure 6b. The distribution clearly proves that the majority of the orientations lie near a Pitsch variant. The orientation density maximum is found at 0.88° of misorientation and is therefore larger than expected for a maximum resolution of 0.5°. A part of the deviation with respect to a Pitsch variant is attributed to the slight crystal orientation spread in the BCC grain, which was determined to be about 0.3° from the uncorrelated misorientation distribution inside one BCC grain. These two deviations together explain the maximum position around 0.88°.

## Discussion

The EBSD data reveal detailed features in the crystal orientation distribution. Together with a detailed analysis of the microstructure, we will explain and discuss the OR in relation to the mechanisms for nucleation and grain growth of FCC Widmanstätten plates in Al_0.7_CoCrFeNi. Indeed, the combination of the Widmanstätten plates, the well-defined orientation distribution, and the two-phase microstructure make this Al_0.7_CoCrFeNi an attractive material to study the BCC to FCC phase transformation.

The two main features of the crystal orientation distribution are the splitting of the {111}_FCC_ pole distribution, which are parallel to the {110}_BCC_ poles, and the continuum of orientations between a Pitsch variant and K–S variant.

**Figure 7:**
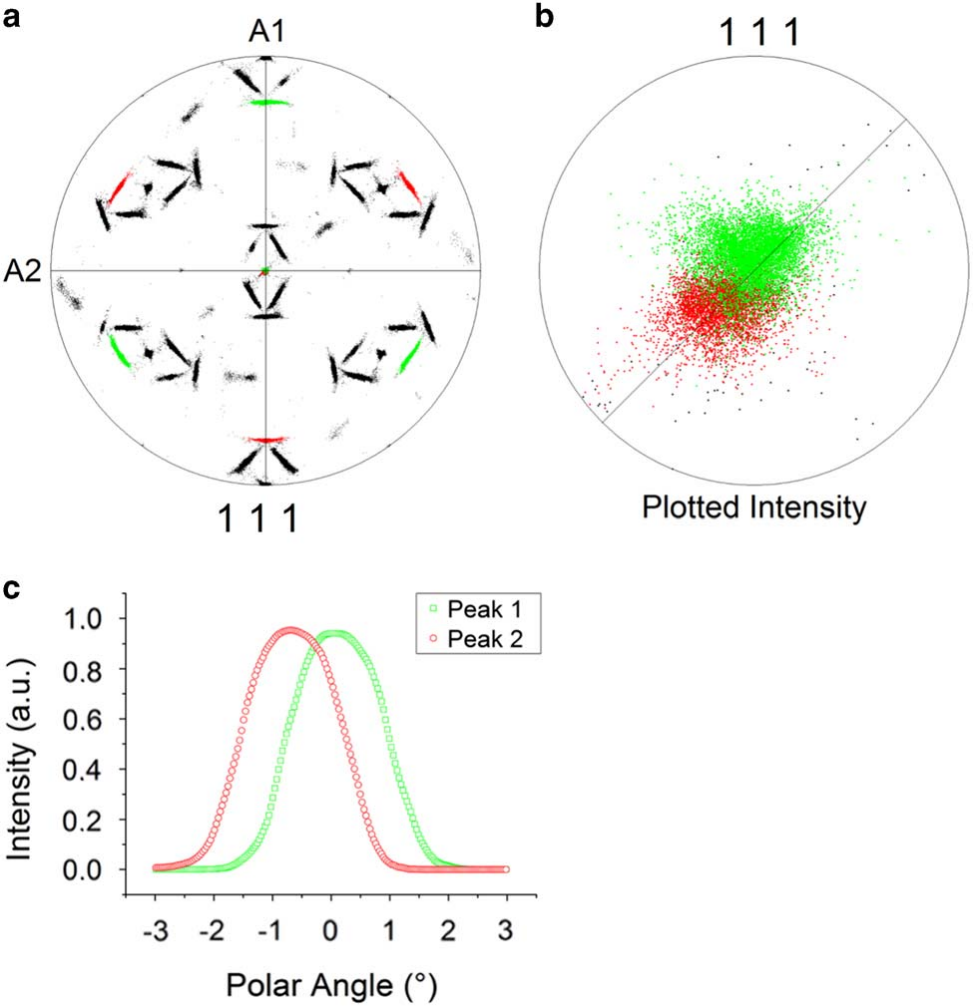
Shift of the {111} pole peaks. **a:** Observed separation of the theoretical {111} pole distribution of two Pitsch variants. **b:** The magnified pole figure with maximum angular distance of 15° shows two pole clusters with some overlap. **c:** The pseudo-rocking curve shows a similar distribution for both clusters and the peak shift from the center of 0.38°±0.01°.

Initially, the OR for this BBC to FCC phase transformation is determined as the Pitsch OR – i.e., {111}_FCC_ ||{110}_BCC_ and <011>_FCC_ ||<001>_BCC_ – however, a more detailed analysis reveals a discrepancy between the theoretical Pitsch OR and the OR found here. A misfit of 0.46±0.01° was already observed between the centers of the pole distribution and the Pitsch variants in Figure 3; moreover, we also observed no common {111}_FCC_ plane for two variants in the HEA, whereas according to the Pitsch OR a common {111}_FCC_ plane parallel to a {110}_BCC_ plane should be present for these two variants. To visualize the discrepancy, the data were rotated such that a {111}_FCC_ pole cluster is parallel to sample axis A3 and each of the two Pitsch variants have been given a color; Figure 7. It is clearly visible that the {111}_FCC_ pole clusters of these two Pitsch variants do not overlap, but are slightly shifted from each other. Our analysis confirms that this discrepancy holds for all the {111}_FCC_ pole clusters. The pole distribution along the 45° line in Figure 7c quantifies the shift of both similar-shaped pole clusters at 0.38±0.01° with respect to the center.

The second characteristic feature is a continuous set of varying orientations between a Pitsch and K–S variant. The pole density decreases with a rotation toward a K–S variant as is seen in Figure 5b and 6b. Both distributions show a dominant peak near the Pitsch variant. In terms of Euler angles a small difference between the theoretical Pitsch variant and the local maximum is measured; see Table 4. Moreover, we observe in Figure 6a clusters of a Pitsch variant in all grains, whereas clusters of a K–S variant are only observed in larger, thicker grains. This fact, together with the assumption of the same nucleation mechanism for all plates and with a predominance of Pitsch OR observed in distributions shown in Figures 5b and 6b, lead to a conclusion that the Pitsch OR is the most favorable OR during nucleation. The character of the experimental OR distribution is one particular dominating OR (Pitsch), which gradually rotates to another one (K–S). This character is different from a case when a simple combination of two or few ORs was detected (He et al., 2006; Verbeken et al., 2009).

A continuous variation of orientations between the classical OR variants of K–S, N–W, Pitsch, and Bain were mentioned before in the literature and various terms were assigned to these phenomena, such as “Visible Transition Path” (Nolze & Geist, 2004) and “Continuity between orientations” (Cayron, 2013). The origin of these variations of orientations were brushed aside as an artifact introduced by low signal–to-noise ratio in crystal orientation measurements, or due to the inhomogeneity of orientations within a grain (Verbeken et al., 2009). Further, an OR with more than 24 cubic variants is suggested, which gives more irrational (Nolze, 2008) or hybrid ORs in comparison with the commonly accepted ones. Cayron (2013) explains the continuum of orientations seen in the pole figures using a one-step model for the martensitic FCC to BCC phase transformation. In this one-step model, the spread in misorientation is due to a distortion introduced by differences in lattice parameters of the (110)_FCC_ plane in the (111)_BCC_ plane during grain nucleation according to the Pitsch OR. This is called a Pitsch distortion. The newly formed martensitic grain is nucleated with the Pitsch OR and upon nucleation distorts the surrounding lattice of the parent grain. The further growth of a FCC daughter grain is therefore distorted by a small angle with respect to the initial nucleation. Cayron (2013) showed that the continuous distributed orientations are closing the K–S close-packed directions through the Pitsch OR. Due to the Pitsch distortion, a symmetric gradient in orientation rotation with respect to a Pitsch variant is therefore expected in the daughter phase as a function of the thickness during its growth. The continuous variation of orientations between the different theoretical ORs (K–S, N–W, Pitsch) can be described as a single rotation with a maximum angle of 5.26°. The orientations between Pitsch and K–S can therefore be described by a rotation around the [111]_BCC_ direction with an angle between 0 and 5.26°; the orientations between K–S and N–W can be described by a rotation around (110)_BCC_ plane normal with an angle between 0 and 5.26°; however, a lattice cannot convert directly from a Pitsch variant to an N–W variant. The Pitsch variant first needs to rotate to a K–S variant around <111>_FCC_. This is followed by a rotation from the K–S variant to the N–W variant along the <110>_FCC_ direction. These two separate rotations match with the rotations going from one OR to another already described using the in-line invariant method described by Dahmen (1982).

In the microstructure of the HEA, a gradient in orientation is found, but the gradient is not symmetric. We exclude the possibility that a difference in elemental composition is the cause of the difference in OR. EBSD in combination with EDS was performed and the elemental composition was compared with the rotation angle from the Pitsch variant to the K–S variant. The comparison shows no correlation between the misorientation angle with respect to a Pitsch variant and the elemental composition.

Both the discrepancy as well as the continuous variation of orientations can be related to the differently sized atoms present in the HEA. Although the BCC parent phase was indexed as a disordered A2 phase it also contains an ordered B2 phase (Rao et al., 2016; Ma et al., 2017). The B2 lattice parameters differ locally depending on the spread in different sizes of the constituting atoms. Therefore, the surrounding lattices are strained and deviate from a perfect cubic structure. The first consequence of differently sized atoms is that the lattices are not perfectly cubic but tetragonal and, therefore, the Pitsch OR does not hold completely (Muehlemann & Koumatos, 2016). The following correction should be made for the tetragonality of the phase according to Muehlmann and Koumatos in the parallel plane description of the Pitsch OR:
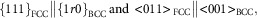



with *r* being the ratio of lattice parameters c/a of the tetragonal symmetry. The description reduces to the original Pitsch OR if the lattice is cubic. From the {111}_FCC_ pole splitting in Figure 7 and the deviation of the {001}_FCC_ pole distribution center in Figure 3, the average tetragonality ratio can be calculated for the HEA and it ranges between *r*=1.013 and *r*=1.016. The second consequence of differently sized atoms is that the lattice parameters vary locally, which affect the fit for the planes at the interface. From an invariant line point of view, the lattice parameter ratio dictates the relative orientation between a {111}_FCC_ and {110}_BCC_ plane (Dahmen, 1982) and, therefore, the OR in this case ranges from the Pitsch to K–S OR. The mean lattice parameters determined by X-ray diffraction (Ocelík et al., 2016) for the equilibrium BCC phase and FCC phase for this type of HEA are 2.899 and 3.596 Å, respectively. The lattice parameter ratio *a*_FCC_/*a*_BCC_, therefore, is 1.240. According to Dahmen, rotations of around 5.26° with respect to Pitsch are favorable for this lattice parameter ratio and therefore the exact K–S OR is most favorable; however, we observe a majority of Pitsch OR in our HEA. From an interfacial energy point of view the preferential nucleation and interfacial OR are related to the ratio of atomic radii (Gotoh et al., 1988). Gotoh et al. investigated the preferential epitaxial OR at (110)_BCC_||(111)_FCC_ interfaces. This is exactly the Al_0.7_CoCrFeNi case: the continuum of orientations within a Pitsch to K–S pair satisfy the (110)_BCC_||(111)_FCC_ condition. As Al_0.7_CoCrFeNi is a compound and consists of both ordered B2 phase and disordered A2 phase, the radius varies considerably and the elemental composition may very locally be inhomogeneous. From the mean lattice parameters, an “apparent” atomic radius can be calculated from geometrical arguments and, therefore, the mean ratio of atomic radii as follows:1
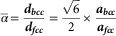
with ***α*** is the atomic radius ratio, ***d*** the atomic radius, and ***a*** the lattice parameter. From equation (1) it follows that the Al_0.7_CoCrFeNi HEA has a mean atomic radius ratio of 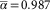. According to Gotoh et al. (1988) the Pitsch OR is favorable for 0.83<*α*<0.88 and 1.02<*α*<1.19 and a K–S OR is favorable for 0.88<*α*<0.96. The mean atomic radius ratio of 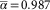 therefore does not fit within any specific intervals suggested by Gotoh et al.; there is no preference for either the Pitsch or the K–S OR. From equation (1), depending on the lattice parameter ratio (Dahmen, 1982) and the atomic radii of Al, Co, Cr, Fe, and Ni, the range of rotation of the {111}_FCC_ with respect to the {110}_BCC_ plane can be calculated. Given the atomic of radii of the largest and smallest element, *d*_Al_=143 pm and *d*_Ni_=124.6 pm (Greenwood & Earnshaw, 1997), one calculates that the range of lattice parameter ratio is from 1.067 to 1.406. According to Dahmen this includes the full range of rotation from 0 to 5.5°. Therefore, not only a Pitsch or K–S OR are found at the interface, but a continuous set of orientations between Pitsch and K–S. Similarly, this is also true for the analysis described by Gotoh et al. The fact that the distribution in Figure 6b is not homogenous hints to the fact there is a preferred lattice size due to the differently sized atoms and inhomogeneity in composition.

The local inhomogeneity of atom distribution introduces a strong local lattice distortion in HEA, which is possible because of the ordered and disordered phases within the BCC phase and, therefore, influences the orientation distribution. This property locally breaks the symmetry by forming tetragonal lattices. Thereby the parallel planes are shifted and local variations in lattice parameters are introduced. These contributions lead to the rotation on the interfaces.

The morphology of the transformation product highly depends on the OR in the HEA. The phase transformation is a solid–solid transformation with minimum diffusion, as supported by the high cooling rates. The average sample composition corresponds to the nominal composition of the alloy – see Table 3 – however, a slight difference in composition was detected with EDS between the BCC phase and the FCC phase. The BCC phase contains relatively more Al and Cr and less Fe, Co, and Ni atoms than the FCC phase; however, this difference in composition is significantly smaller than the difference in homogenized samples in which the Al composition of the BCC phase and FCC phase is ~33 and ~14 at% Al, respectively (Kao et al., 2009; Ocelík et al., 2016; Zhang et al., 2016 *b*). For the homogenized HEA, an Al-rich BCC phase is solidified during casting and enriched by diffusing Al atoms from the FCC phase during homogenization, thereby creating an Al-depleted FCC phase. The phase transformation during casting does not occur on a solid–liquid interface at which the solidification phase is changed because of a change in elemental composition in the liquid and moving solidification front.

**Figure 8:**
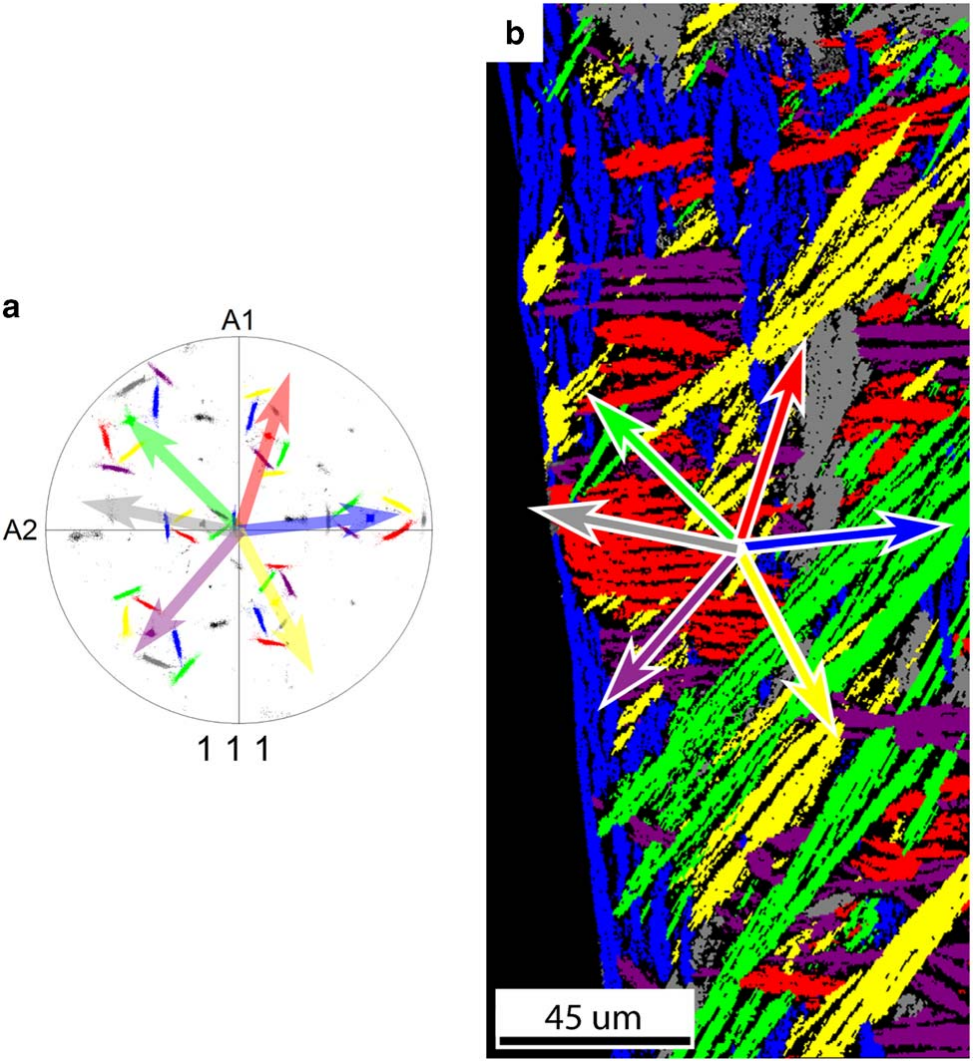
**a:** The {111} pole figure of the FCC grains’ origination from one parent BCC grain; 12 Pitsch variants exist, which are grouped in six variant pairs of two variants with a common <111>_FCC_ direction. All orientations belonging to a variant pair are given the same color. The common <111>_FCC_ direction of each pair is parallel to a <110>_BCC_ direction. The arrows depict the projection of the common <111>_FCC_ direction of each variant pair. **b:** Grain map of the FCC map with colors corresponding to the {111} pole figure. The arrows depict the projection of the common <111> direction of each variant pair corresponding to the pole figure. A correlation has been found between the <111> direction projections and the minor axis of the grain. A small deviation has been found for the purple and red grains.

The FCC daughter plate morphology correlates strongly with the crystal orientation of the BCC parent grain. From all 12 Pitsch variants there are six variant pairs of two variants with a near common <111>_FCC_ direction. Each of the six variant pairs are colored differently in the pole figure in Figure 8a. The grains corresponding to the variant pairs are found in the map in Figure 8b, which was constructed from the pole figure by highlighting points with the same <111> direction with corresponding color with a 5° tolerance angle.

A number of things are observed comparing Figures 8a and 8b. First, each plate belongs to one of six variant pairs represented by six different colors. Second, if we compare the projection of the common <111>_FCC_ directions of each variant pair in the pole figure with the direction of the minor axis of the corresponding plate shape, we observe that they are parallel; however, for red and purple grains a small deviation between the {111}_FCC_ plane normal and the minor axis of the grain is observed.

**Figure 9:**
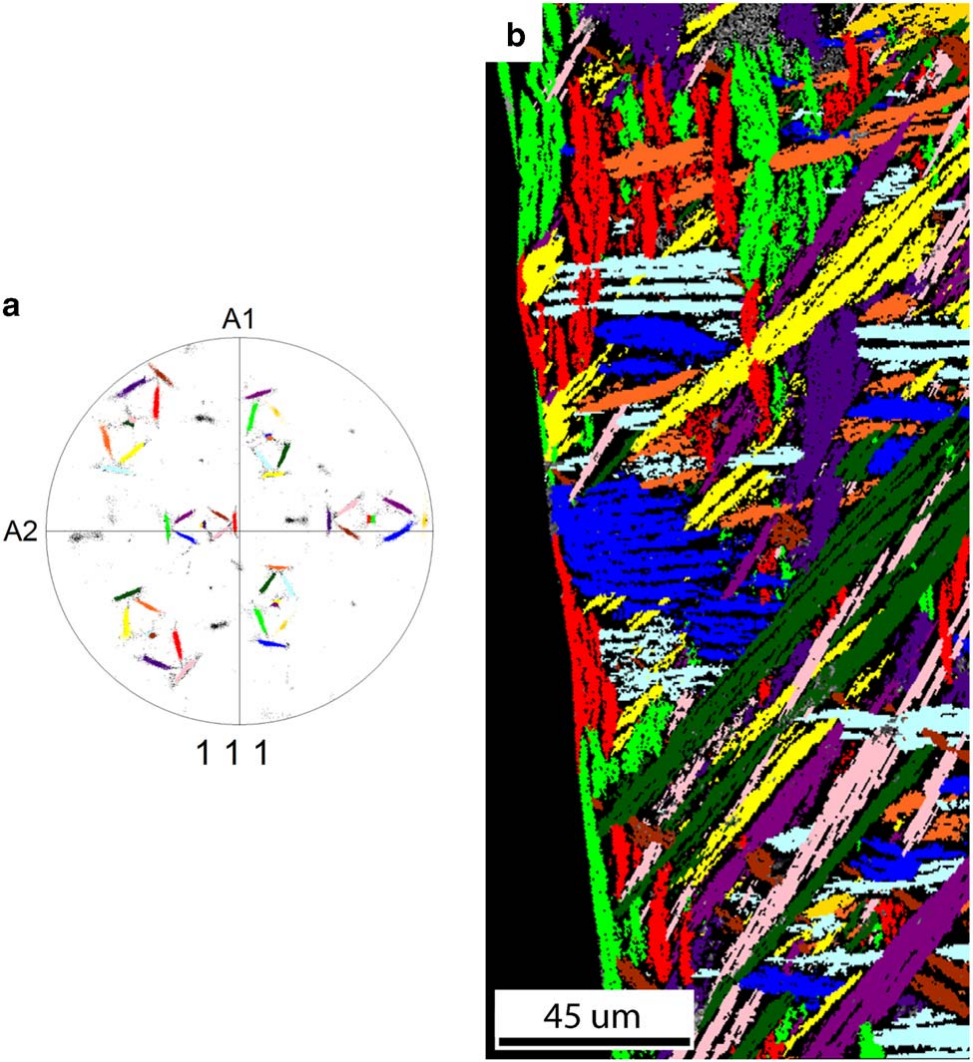
**a:** The {111} pole figure shows the 12 separated {111} pole clusters, each labeled with a unique color. Each of the 12 clusters belongs to a Pitsch variant plus rotation to two Kurdjumov–Sachs variants. **b:** The grain map of the FCC phase shows that the grain shape orientation of grains from a variant pair slightly differ from each other. The colors in the grain map correspond with the colors in the {111} pole figure.

If we take the separation of the common <111>_FCC_ direction in each variant pair into account, we observe a number of additional features, shown in Figure 9. In this case 12 groups can be distinguished corresponding to the 12 Pitsch variants plus a rotation around the split <111> direction toward a K–S variant. Further, in Figure 9, the 12 colors of the pole figure and grain map correspond to each other. First, each grain only consists of orientations from one Pitsch variant plus a K–S pair; i.e., one Pitsch variant and orientations with a maximum misorientation of 5.5° along the <111> direction. Second, there is a slight difference between the grain shape orientation within a variant pair; for instance the dark green and pink plates. This difference is best visible when the projections of the common <111> direction in one variant pair in the pole figure create a large angle. This difference corresponds to slightly differently orientated plates in the bulk. Finally, two variants belonging to the same variant pair are always in the vicinity of each other, having a misorientation close to Σ3 (60°@(111)), and they probably have the same nucleation origin.

A particular reason why Pitsch nucleation is favorable in Al_0.7_CoCrFeNi has not been found. In the literature, different cases for the BCC to FCC phase transformation are presented. Each material may show its specific combination of nucleation OR and OR continuum. In low-carbon steels the K–S OR nucleation is observed and the orientation gradually changes to a Pitsch variant (Cayron, 2014); however, duplex steel proved to be useful for understanding ORs in steel, in which FCC precipitations nucleate in a BCC matrix (Ohmori et al., 1995; Redjaïmia & Metauer, 2001; Jiao et al., 2003; Qiu & Zhang, 2007) and the K–S OR was predominantly found. In Fe–Mn–Al alloys a K–S OR and a near-Pitsch OR were found for FCC precipitates in a BCC matrix (Cheng & Lin, 2002). Moreover, the Widmanstätten plates in the microstructure are not linked to a specific combination of nucleation and growth. Bunge et al. (2003) characterized iron meteorites containing Widmanstätten plates using synchrotron X-ray radiation and observed a continuous variation in orientations between a K–S and N–W pair. In addition, in other iron meteorites, characterized by SEM+EBSD, a dominant KS and NW OR is observed (Nolze & Geist, 2004; Goldstein & Michael, 2006); however, the Watson meteorite characterized by SEM+EBSD had a Pitsch and G–T OR (Nolze et al., 2005).

## Conclusions

In conclusion, we may state that EBSD observations provide considerable statistics for OR between the FCC phase and BCC phase, which could be used for a precise orientation estimation based on an assumption of Gaussian error distribution in crystal orientation measurements. The EBSD technique allows us to sample with far greater numbers of orientations in comparison with TEM, resulting in better statistics in OR studies. We have investigated the crystal orientation distribution in great detail and the results are correlated to the phase transformation, nucleation, and grain morphology in Al_0.7_CoCrFeNi-cast HEA. We can conclude the following:

∙ We found a continuum variation in orientations ranging from the Pitsch to K–S due to the different sizes of atoms and local composition inhomogeneity due to the ordered B2 phase. This continuum originates from the Pitsch OR-based nucleation.
∙ The difference in atom sizes inherent to the HEA is the cause for the splitting of two Pitsch variants with a common {111} plane. The tetragonal ratio c/a has been found experimentally to be between 1.013 and 1.016.
∙ The FCC phase transforms out of the BCC phase through a shear transformation with a Pitsch OR-based nucleation and with minimal diffusion.
∙ We observed Widmanstätten patterns in cast HEA after its phase transformation.
∙ The FCC grain plates are oriented parallel to a {110}_BCC_ plane and are, therefore, a product of the parent grain orientation, the nucleation OR, and the shear transformation. The tetragonality of the BCC phase has an effect on the grain orientation.
